# Small-Format Drug Conjugates: A Viable Alternative to ADCs for Solid Tumours?

**DOI:** 10.3390/antib7020016

**Published:** 2018-03-30

**Authors:** Mahendra P. Deonarain, Gokhan Yahioglu, Ioanna Stamati, Anja Pomowski, James Clarke, Bryan M. Edwards, Soraya Diez-Posada, Ashleigh C. Stewart

**Affiliations:** 1 Antikor Biopharma Ltd., Stevenage Bioscience Catalyst, Gunnels Wood Road, Stevenage Herts SG12FX, UK; g.yahioglu@antikor.co.uk (G.Y.); i.stamati@antikor.co.uk (I.S.); a.pomowski@antikor.co.uk (A.P.); j.clarke@antikor.co.uk (J.C.); b.edwards@antikor.co.uk (B.M.E.); s.diez-posada@antikor.co.uk (S.D.-P.); a.stewart@antikor.co.uk (A.C.S.); 2 Department of Chemistry, Imperial College London, Exhibition Road, London SW72AZ, UK

**Keywords:** antibody–drug conjugate, fragment, alternative scaffold, penetration, pharmacokinetics

## Abstract

Antibody–Drug Conjugates (ADCs) have been through multiple cycles of technological innovation since the concept was first practically demonstrated ~40 years ago. Current technology is focusing on large, whole immunoglobulin formats (of which there are approaching 100 in clinical development), many with site-specifically conjugated payloads numbering 2 or 4. Despite the success of trastuzumab-emtansine in breast cancer, ADCs have generally failed to have an impact in solid tumours, leading many to explore alternative, smaller formats which have better penetrating properties as well as more rapid pharmacokinetics (PK). This review describes research and development progress over the last ~10 years obtained from the primary literature or conferences covering over a dozen different smaller format-drug conjugates from 80 kDa to around 1 kDa in total size. In general, these agents are potent in vitro, particularly more recent ones incorporating ultra-potent payloads such as auristatins or maytansinoids, but this potency profile changes when testing in vivo due to the more rapid clearance. Strategies to manipulate the PK properties, whilst retaining the more effective tumour penetrating properties could at last make small-format drug conjugates viable alternative therapeutics to the more established ADCs.

## 1. Introduction

There is unlikely to be a better example of multi-disciplinary innovation in oncology therapeutics than in the field of Antibody Drug Conjugates (ADCs). With multiple waves of emerging technologies and their implementation into clinical applications, it is a truly exciting time to be in this R&D space. We have seen many refinements from the original concept, practically exemplified 40 or more years ago, with just about every minute aspect being investigated. These refinements include the payload killing mechanism, linker release action, antibody conjugation position, and the overall chemistry to build an ADC which is maximally effective and well-tolerated in humans. There are now 4 approved products with over 100 ADC candidates in clinical trials [1,2] and even more in preclinical development [2,3], but these are all based on refinements of the same whole immunoglobulin-G (IgG) format. The marketed and clinical pipeline is disproportionately biased towards haematological cancers [4] since treating solid tumours with large macromolecules remains a risk and a challenge [5,6].

**Figure 1:**
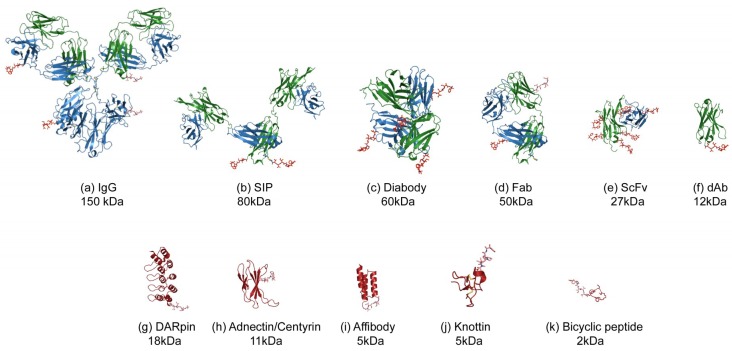
Illustration of the various formats for Antibody (Fragment) or alternative scaffold–drug conjugates. The protein or peptide formats are drawn to scale with an MMAE payload using published Protein Database files (https://www.rcsb.org) as follows: (**a**) IgG-DAR4, 1igy [12], (**b**) SIP-DAR2 (modelled on 5c2b [13]), (**c**) diabody-DAR4, 5fcs [14], (**d**) Fab-DAR2, 5n2k [15], (**e**) scFv-DAR8, 5c2b [13], (**f**) dAb, VH domain-DAR1, 5tsj [16], (**g**) DARPIn-DAR1, 4j7w [17], (**h**) Adnectin-DAR1, 3qwr [18], (**i**) Affibody-DAR1, 1lp1 [19], (**j**) Knottin-DAR1 1w7z [20], (**k**) Bicycle-DAR1, 5i8m [21]. The payload used for illustrative purposes is MMAE, 5iyz_4q5 [22] which is hypothetically conjugated to surface residues such as lysines (IgG, Fab, scFv) or C-terminal residues such as cysteine (SIP, Diabody, dAb, alternative scaffolds). The molecular weights indicated are for the protein without payload.

This review will focus on an emerging area [7] where smaller formats are being used to deliver the cytotoxic payload. The superior tumour penetration and rapid systemic clearance properties [8,9] offer an alternative and potentially a wider therapeutic window than the larger ADCs, which take days to reach peak accumulation and weeks to clear from the body [10,11]. Unlike ADCs which have well understood pharmacokinetics (PK) it is not known what the ideal PK profile will be for a small-molecule, peptide, small binding protein or antibody fragment–drug conjugate. What is clear is that the payload will have a greater impact on this PK profile as well as the biophysical properties such as aggregation and target binding, due to it being a more dominating feature of the conjugate complex (Figure 1). Because of this, the design of the linker payload will need special consideration.

The focus of this review will be therapeutic drug conjugates, smaller than the IgG-based ADC formats where the payload is a small cytotoxic molecule, with toxin fusion proteins and radio-immuno-conjugates excluded. More general topics such as ADC and payload action will not be covered as they are reviewed adequately elsewhere (for examples [3,5,7,23] and within this special issue.

## 2. Uptake and Tumour Penetration of Smaller Binding Formats

Antibody uptake is a complicated issue, made even more problematical when considering ADCs as the payload being delivered is an active cytotoxic. There are many excellent practical [24,25] and theoretical papers covering this topic [26,27]. Total antibody uptake must be considered separately from tumour penetration. If an ADC-payload does not access and kill all parts of the tumour mass, therapy will be poorly effective despite high overall uptake. Perhaps worse, zones of marginal cytotoxic drug concentrations could foster drug resistance rendering therapy completely ineffective [23,28].

Tumour targeting of a systemically administered antibody involves 4 major steps. Blood flow to the tumour, transport across the capillary wall (extravasation), diffusion through the tissue and binding/internalisation at the cell surface [26]. Blood flow can be 100–1000× faster than the slowest of these steps, extravasation, meaning that the ADC concentration at the perivascular boundary of a tumour can be 2–3 orders of magnitude lower than the bulk plasma concentration [29]. The high interstitial pressure within solid tumours hinders local convection meaning that the main method of transport through the tumour is diffusion [26]. Extravasation and diffusion are faster for smaller molecules (leading to a larger volume of distribution [8]) compared to large antibodies but the long half-life of IgG helps to deliver more absolute antibody to the tumour. Whole IgG has a slower target-independent clearance rate (days compared to hours/minutes for smaller formats) due to salvage recycling by the neonatal Fc receptor (FcRn) pathway and exclusion from renal filtration (cut-off ~60–70 kDa). This latter point was practically illustrated by Adams et al. [24], supported by modelling from Thurber et al. [26], that the uptake for single-chain variable fragments (scFvs) can approach that of IgG in nephrectomised mice, due to the impaired renal clearance.

Tumour penetration and micro-distribution is another complicated topic as it is influenced by many factors including dose/systemic clearance, antigen density, extra-cellular matrix structure, receptor internalisation rates, binding affinity, diffusion rate/coefficient and format biophysical size (hydrodynamic/Stokes’ radius) [30]. Modelling of ADCs [31] and antibodies [26,29] found that high receptor levels hindered penetration due to the target mopping up the ADC diffusing from the capillary. Modelling cytotoxicity has suggested a direct reduction in tumour killing potency and that 10^4^–10^5^ receptors/cell was seen as the maximum [31]. Consistent with this, slower internalisation rates are needed for highly expressed receptors as this allowed better penetration leading to better efficacy. Monovalent binders, which typify most of the antibody fragments, and alternative scaffolds may help here by having reduced internalisation rates due to no cross-linking. The reverse was seen for low receptor expression. High affinity is important for low receptor expression but anything stronger than 10 nM *K*_D_ (equilibrium dissociation constant) was not favoured for highly-expressing receptors, leading to a front of bound antibody within a tumour with little penetration beyond it (‘binding site barrier’) [32,33]. 

Practical [32,34,35] and theoretical [27,29] studies support the concept that due to their smaller physical radius and hence faster diffusion and extravasation coefficients, antibody fragments and smaller formats penetrate tumours more rapidly than immunoglobulins. But the trade-off is lower overall uptake due to other major factors such as systemic clearance and endocytic/tumour catabolism, meaning that solid tumours never reach saturation with targeted drug, which is important for cure efficacy [36]. 

For cytotoxic therapies, tumour penetration and killing potency is only half of the equation. Tolerability and adverse effects need to be minimised if the required concentrations needed for efficacy are to be obtained in patients. This is where smaller formats can make a difference. The lack of an Fc domain reduces cross-reactivity with Fc-receptors on various normal cells, and the lower overall plasma exposure to normal tissues reduces the likelihood of the payload decoupling and intoxicating non-target cells. These factors can help to increase the overall therapeutic window which for solid tumours needs to be at least 10-times larger than in haematological cancers to drive the penetration deep into the tumour [37].

As demonstrated with scFvs, high affinity restricts tumour uptake due to the binding site barrier effect, with the optimal affinity for high overall percentage uptake being 1.0–0.1 nM for HER2 [24]. However, lower affinities resulted in more even penetration. Primarily, perivascular binding was seen with a 10 pM affinity anti-HER2 scFv whereas 4-times better penetration was seen for lower affinity scFvs [32]. In another example, an 8 nM affinity gave more even penetration than 30 pM for CEA scFvs at high doses [29]. 

## 3. Antibody Fragment–Drug Conjugates

When considering smaller formats of ADCs, antibody fragments are an obvious choice. These can be potentially more stable, especially fragments that do not contain the more thermo-labile CH2 domain. Many ADC patents include claims relating to antibody fragment drug conjugates (FDCs) however, without practical examples. Fragment antibody (Fab) fragments can have the remnant of the IgG hinge region leaving a natural thiol/disulfide conjugation point, but more often than not, C-terminal cysteines are specifically engineered, following the trend with IgG site-specific conjugation strategies [3,5]. Thiol or disulfide re-bridging technologies for conjugation are well established and extensively reviewed elsewhere [38]. Next-generation maleimide linkers have addressed the instability seen with thioether bonds in early generation ADCs. Glycan conjugations are not usually followed due to the primarily prokaryotic manufacturing systems used for antibody fragments, but non-natural amino acid incorporation and enzymatic conjugation approaches are still very applicable [39].

### 3.1. Fab–Drug Conjugates

The Fab format has largely been superseded by recombinant single-chain formats such as the scFv, diabodies (head-to-tail dimer of a scFv), and single domain antibodies. There have been reports of Fab–Drug Conjugates with low potency payloads such as paclitaxel [40] or doxorubicin [41] which have micromolar to low nanomolar potency. These values would be ineffective for an ADC with higher uptake, so it was no surprise that these were also poorly efficacious. Using a current-generation MMAE payload, an anti-HER2 trastuzumab Fab drug conjugate had sub-nanomolar potency in vitro but required alternate day dosing at 20 mg/kg to achieve any sort of tumour growth control [42]. A Trop2-targeting Fab-doxorubicin conjugate, of unknown drug to antibody ratio (DAR) had a high nM potency and modest in vivo efficacy when given at 6 mg/kg every two days (equivalent to 2 mg/kg doxorubicin payload) [43], conjugated via a maleimide linker to the Fab thiols.

An anti-CD20 Fab with two residual hinge thiols was conjugated to a homogenous DAR 2 using doxorubicin containing sizable (2 kDa) PEG chains. The in vivo pharmacokinetic impact was not investigated, but presumably these PEG chains were needed to overcome the high hydrophobicity of the payload. As expected these Fab drug conjugates were not very potent (only 20–30% cell kill at 10 μg/mL, approximately 200 nM range) resulting in a modest reduction in tumour growth in vivo (1 mg/kg equivalent of doxorubicin). Although all the animal body weights deteriorated in the study, the conjugate was better tolerated [44].

Developing a protein-A based solid phase conjugation method, Puthenveetil et al. [45] produced a trastuzumab Fab drug conjugate, dual labelled with a cleavable and non-cleavable auristatin-based payload (overall DAR 3). This had an in vitro potency of 0.7–0.9 nM (37–43 ng/mL), similar to the Fab drug conjugate described by Badescu et al. [42] and likely to have similar weak in vivo efficacy.

The most potent Fab drug conjugate to date, with an EC_50_ of ~100 pM acts as a bispecific antibody re-targeting cytotoxic T-cells, rather than a conventional ADC [46]. Here, the payload was dicarboxypropyl ureido pentanedioic acid (DUPA), a small-molecule ligand selective for prostate-specific membrane antigens. Interestingly, the half-life of the conjugate was extended to around 5-times the typical 1 h half-life of the unmodified Fab, resulting in a good PK profile for efficacy especially compared to other small format T-cell engagers of similar molecular weight. This was attributed to the increased hydrophobicity, although no biodistribution data was shown.

### 3.2. Diabody–Drug Conjugates

Kim et al. [47] provided the first comprehensive analysis of a potent antibody fragment drug conjugate using an anti-CD30 diabody with 4 cysteine thiols conjugated to MMAE and MMAF payloads bearing maleimide linkers. The diabody ADC (using MMAF, which was 4 to 7-fold more potent than MMAE) with a DAR of around 4 was compared to an equivalent IgG ADC. Hence, the two formats had comparable DARs and valency. The whole antibody-based ADC was around 4-fold more potent in vitro but around three times more diabody drug conjugate dose was needed to match the ADCs in vivo efficacy (7.2 vs. 2 mg/kg). Crucially, the diabody–drug conjugate had a faster blood clearance reflected by its smaller size, but the 30-fold lower exposure level only led to a 3-fold drop in efficacy. The renal clearance expected of such fragments was not evident, suggesting that the payload had a major influence leading to a re-distribution of conjugate to the liver.

Commercially, diabody-like formats are being developed as ADCs, notably Avipep’s ‘Avimers’ [48]. DARs of 2 and 4 have been obtained on engineered diabodies. Drug candidates such as AVP10 (lymphoma) are being developed, but the Company’s lead product is a radio-labelled Avimer AVP04.

### 3.3. SIP–Drug Conjugates

Neri and co-workers have had a great deal of success developing anti-tumour vasculature scFvs and small immuno-proteins (SIPs-scFvs dimerised using a CHε4-domain, therefore approximately half the size of an IgG) against fibronectin and similar targets [49,50,51,52,53,54]. Tumour vascular targets like these are more accessible, stable, and common to many tumour types. The SIP format is favoured due to higher tumour uptake compared to smaller antibody fragments, but has greater specificity/contrast compared to IgGs. The incorporation of two C-terminal cysteines allows payload conjugation using disulfide and other chemistries [49,50]. 

There are not many examples of ADCs targeting non-internalising antigens as the generally accepted view is that the conjugate needs to be internalised for the payload to be efficiently released to reach the DNA or tubulin target. Neri’s approach for anti-vasculature targeting using high-affinity antibody fragments works well as there are no penetration issues, but the mechanism is subtly different with extracellular payload release and complete vasculature destruction leading to tumour regression. The C-terminal disulfide on the F8 anti-EDA (fibronectin) SIP allowed direct coupling of thiol-bearing payloads that have a ‘traceless’ linker. Their observation was that the more exposed the thiol was, the less stable the ADC was, supporting other thiol-positioning and site-specific conjugation ADC ideas [50]. 

Using a moderately potent payload, cemadotin, ADCs were made that caused tumour growth delays at 43 mg/kg [49]. In this mechanism, dying cells release reducing moieties such as GSH and Cys, which can reduce the disulfide of the linker-payload, releasing the payload to diffuse into the tumour cell. An amplification of this effect happens as more cells die. The bystander effect of killing tumours without targets is likely to be stronger if the drug is released outside the cell. The F8 antibody was also conjugated to the more potent DM1 payload with well-tolerated cures being observed at three doses of 7 mg/kg given three times [51]. 

Another non-internalising antibody, F16 (anti-tenascin-C) was conjugated to cleavable valine-citrulline-MMAE (vcMMAE) via engineered cysteines. The resulting SIP drug conjugate with a DAR 2, was not as stable as the IgG equivalent in serum and this was further supported by a therapy in tumour bearing mice where the IgG significantly outperformed the SIP drug conjugate dosed four times at 7 mg/kg [52]. However, this approach showed that payloads could be proteolytically released in the extra-cellular space, further opening up the opportunity for targeting solid tumours via easier to access stromal/vasculature targets. A further innovation was the incorporation of N-terminal cysteines (C-terminal was also possible), which generates a 1,2-aminothiol that can be conjugated to aldehyde-bearing payloads forming a thiazolidine linker. Using cemadotin, nanomolar potency ADCs were obtained using an F8 diabody [53]. 

A side-by-side comparison of IgG vs. SIP antibody fragment format was made using the F8 antibody and DM1 payload conjugated at a DAR 2 as a C-terminal disulfide [54]. As expected, the SIP–drug conjugate accumulated into the tumour and cleared more rapidly and the 24 h uptake levels were more than 4-times higher for the IgG ADC. The payload on the ADC was at least 10-times more stable, but the SIP conjugate was more effective even on a molar basis. The authors attributed the faster drug release leading to higher tumour payload exposure over a shorter period of time (rather than slow-release as seen with an ADC) being the reason for the better performance [54].

Neri has also combined their ADC approach with their more advanced immunocytokine antibody fusion proteins to generate trifunctional antibodies (F8 diabody-IL2 fusion protein with a cysteine in the linker for thiol payload conjugation) that have an immunostimulatory function as well as being an ADC. The synergistic behaviour was thought to be due to dying cells having increased immune-stimulatory power. This is called an IDC (immunocytokine drug conjugate). This format was exceptionally potent at around 0.5 mg/kg in an F9 teratocarcinoma model [55]. 

### 3.4. scFv–Drug Conjugates

Single-chain Fvs are a versatile fragment format, arguably the corner-stone of many antibody discovery programs. However, even with a DAR as high as 14:1, achieved by conjugating onto oxidized dextran [56], a scFv-adriamycin FDC had potency in the low nM range and modest efficacy in vivo suggesting a low delivery rate.

Targeted photodynamic therapy (T-PDT) can be considered a form of ADC where the payload (a photosensitiser) needs to be further activated using light (usually a laser). Quite often, antibody fragments are used to reduce systemic exposure before laser illumination takes place. Such light-activated ADCs could have reduced side-effects compared to conventional ADCs as the cytotoxicity is separated from the tumour targeting. Our team spent many years developing this approach, carefully matching the linker-payload to the format for optimal conjugation [57,58]. However, despite compelling in vivo data (tumour cures at 4 mg/kg in a HER2 model), this could not be translated into a viable oncology drug candidate. Boyle, in collaboration with Neri’s group, also generated strong data with evidence of immune-cell involvement in the therapeutic efficacy [59].

One advantage of T-PDT is that the photosensitiser can have other useful optical properties that can be used, for example in imaging. These so-called combined therapeutic-diagnostic agents (‘theranostic’) could have the dual purpose of imaging tumour location as well as destroying them. IR dyes have been favoured for this approach. An anti-EGFR scFv appended with a SNAP tag was site-specifically coupled to an IR700 dye (DAR 1) to give a visible fluorescent conjugate with photo-immunotoxicity values in the 45–66 nM range for EGFR-expressing ovarian cancer cell lines [60]. Where the photosensitizer had an emission spectrum unsuitable for imaging, dual payload conjugates onto antibody fragments (photosensitizer and imaging dye) have been described [61].

Through our commercial enterprise, Antikor, we have developed exceptionally potent scFv drug conjugates, branded ‘FDC’ for Fragment–Drug Conjugates. Using ideas about payload spatial separation on a small surface, we discovered that high DAR FDCs, only achievable using surface-exposed lysine residues, were exceptionally potent. The FDCs retained their rapid tumour penetration but had a surprisingly slower plasma clearance rate which was not expected for its small size, still below the renal clearance threshold. DARs of 8–10 were achieved which apparently precluded renal clearance and redistributed clearance to the liver as seen in ADCs. Nanomolar potencies were seen with moderate cytotoxicity payloads such as dolostatin-10 and picomolar potencies were seen with MMAE and MMAF in vitro. This lead to tumour cures at more viable drug dosing regimens than seen before (up to twice per week), whilst still being well-tolerated [62]. More effective tumour penetration was observed compared to comparable ADCs and, despite the high quantity of payload being exposed for a shorter period of time, the high-DAR FDCs were better tolerated [63].

### 3.5. Domain Antibody–Drug Conjugates

In the public domain, antibody fragment–drug conjugates using VH or VH-like domains have been disclosed as pilot projects but have not progressed. This includes companies like Ablynx (nanobody domain antibody technology). Crescendo Biologics Humabody-FDCs comprise ~15 kDa low-DAR conjugates half-life extended using albumin-binding humabodies to retain the benefits of tumour penetration [64].

## 4. Scaffold–Drug Conjugates (SDCs)

The non-antibody binding scaffold field is emerging commercially after many years of R&D. There is only one marketed product, Kalbitor^®^ (ecallantide) (Shire, Cambridge, MA, USA) a kallikrein inhibitor for the treatment of hereditary angioedema discovered from a library of Kunitz domains [65]. However, interest has been growing because they promise to solve the problems posed by conventional antibodies such as expensive manufacturing, glycosylation, formulation, penetration, and thermostability. For example, the type-III fibronectin domain scaffolds Adnectin-BMS and Tn3-MedImmune have melting temperatures of 84–100 °C. These scaffolds tend to be smaller than antibody fragments ranging from 6–21 kDa, can be expressed at high yield in *E. coli*, selected by in vitro display and have higher stability [65]. The majority of applications are for imaging (due to excellent contrast ratios) and receptor/ligand inhibition (VEGF, PD1, CTLA4, IL23, IL6, chymase, and kalkrein, etc.). Only scaffold companies who have disclosed intentions to develop SDCs will be mentioned here.

A recent commercial analysis of this space [66] identified 84 unique alternative scaffold products in development with 82% in preclinical/discovery stage and 40% aimed at oncology. Relatively few (6) were Scaffold–Drug Conjugates (SDCs). 

### 4.1. Affibody–Drug Conjugates

Affibody is a small 6.5 kDa, 3-helix, *Staphylococcus* protein-A, Z-domain derivative with a serum half-life of around 20 min. These are one of the oldest known non-antibody binding scaffolds (reported ~20 years ago) and have predominantly been developed as imaging or anti-inflammatory agents with clinical candidates [67,68]. Substantial protein engineering and phage display has led to binders with low pM affinity [67]. Academic affibody SDCs have been described. For example, one targeting HER2 (ZHER:342) with an Idarubicin payload was described with preliminary in vitro data [69]. A high affinity anti-HER2 affibody (ZHER2891) was conjugated via a site-specifically engineered thiol to releasable vcMMAE (DAR 1). In vitro potencies on high HER2-expressing cells lines were in the low nM range [70]. Half-life extended Affibody-SDCs were made by appending an IgG Fc which also induced dimerisation [71]. The higher affinity species had increased potency in vitro (130 pM) on HER2-expressing SKBr3 cells. No in vivo data has been shown for these types of conjugates.

### 4.2. Fibronectin Type III–Drug Conjugates

Centyrins, a ~100-residue/10 kDa, thiol-free scaffold, are based on the type-III fibronectin domain which can bind to targets with sub-nM affinity with high thermal and chemical stability. Towards making stable Centyrin-SDCs, 94 residues of an anti-EGFR, 100 pM affinity Centyrin were mutated to cysteines and conjugated to a releasable and non-releasable derivative of MMAF at a DAR of 1 [72]. The parental scaffold (83v2) had a Tm of 71 °C and various mutants were discarded due to reduced stability (Tm as low as 46 °C), poor expression and reduced target binding. The two most potent SDCs (N7C and E54C) had an IC_50_ of around 0.2 nM using vcMMAF as the payload, which was ~3× better over the 83v2 SDC with a C-terminal conjugation tag. This was explained by improved folding or proteolytic stability predicted from the accompanying Centyrin X-ray crystal structure. It remains to be seen if these two mutant-enhanced in vitro properties translate in vivo once tumour penetration and uptake pharmacokinetics come into play [72]. A similar conjugate carrying a near infra-red imaging payload (being developed for fluorescence-guided surgery) demonstrated peak tumour uptake after 6–12 h with significant tumour retention after 48 h [73]. No cytotoxic drug–conjugate work has been disclosed on other fibronectin scaffolds such as Adnectins or Tn3-based scaffolds.

### 4.3. Cystine Knot–Drug Conjugates 

Cystine knots are 30–50 residue polypeptides that have remarkable chemical-, protease-, and thermal- stability properties, due to their highly-compact structure [74]. This makes them attractive as delivery vehicles. They also have potential in oral delivery.

Inhibitor Cystine knot proteins are known as knottins. An Integrin-binding, internalising knottin EETI-5F (~1 nM *K*_D_) has previously been demonstrated to have high contrast as an imaging agent with low normal tissue retention. The knottin-SDC strategy used was to incorporate a non-natural amino acid (via solid phase synthesis) bearing an azide chemical group for azide-alkyne conjugation of a gemcitabine payload. Four SDCs were made with various linkers (ester, carbamate, amide and val-ala-PAB). Binding affinity was retained with the releasable peptide linker but the ester linker caused an almost 5× reduction. In vitro potency on U87MG glioma cells was around 8× less potent than the free gemcitabine drug (8.5–9 nM) with the ester linked payload being ineffective. A key observation was that the SDC was able to overcome drug resistance on PANC-1 pancreatic cancer cells, increasing the potency of gemcitabine from 52.8 nM to 2.1 nM. No in vivo data was presented [75].

This work was extended by using cell-free protein synthesis to generate a more ADC-like molecule by appending Fc-domains to the knottin. Copper-free click chemistry was used to attach a more potent MMAF payload for a DAR of 2. IC_50_ values were similar compared to the gemcitabine conjugates (9 nM on U87MG cells), but tumour growth delay was seen in vivo at 10 mg/kg dosing given twice/week for three weeks [76].

A synthetic knottin, chlorotoxin (TM601) was conjugated to the broad-spectrum chemotherapy drug cis-platin (via a succinate link to the free/exposed N-terminal amine). TM601 binds to matrix metalloprotease 2 which is up-regulated in gliomas and related cancers. Conjugation to the 3 ε-amino groups were avoided by carrying out the conjugation reaction at pH 7–8 which favours α-amino groups. Purification and analysis demonstrated a DAR of 1. The chlorotoxin-SDC had micromolar low potency (12–14 μM) that was poorer than that of free cisplatin (2–11 μM), suggesting the payload release was ineffective. Compared to the use of more potent payloads, these conjugates cannot compete with more potent conjugates in clinical development.

Backbone-cyclised cystine knot proteins, known as cyclotides, are also being developed commercially [77] but no drug-conjugates have been disclosed.

### 4.4. DARPin Drug Conjugates

The Designed Ankyrin Repeat class of scaffold proteins are well-advanced with 4 clinical-stage products [78]. These are based on the naturally-occurring ankyrin repeats that can form a continuous binding site whilst displaying high biophysical and protease stability. A high-affinity (129 pM) anti-EpCAM DARPin with a C-terminal cysteine residue for site-specific conjugation was further functionalised with an N-terminal non-natural amino acid azidohomo-alanine for a second conjugation position. This allowed N-terminal attachment of mouse serum albumin as a half-life extension moiety using copper-free click chemistry. C-terminal thiol conjugation of MMAF (DAR 1) formed a DARPin SDC with a potency of around 400 pM for target-expressing cells. In vivo bio-availability was increased 22-fold to around 17.4 h due to the albumin, but the in vivo efficacy benefits were not explored [79].

### 4.5. Abdurin–Drug Conjugates

Unlike most of the alternative binding scaffolds, abdurins retain the ability to bind to the neonatal Fc receptor and have an extended serum half-life. They are a recently-emerging scaffold based on engineered IgG CH2 domains, 1/10th the size (~15 kDa), thermo-stable with 3 loops that can be diversified to form libraries of binders. An anti-ephA2 binder was discovered with 13 nM affinity for the target and 12 nM affinity for human FcRn [80]. Recently, Abdurin-SDC conjugates were described [81] using CyPEG conjugation and vcMMAE payload (DAR 1). Significant loss of target and FcRn binding affinity was observed but moderate in vivo efficacy was seen in PC3 xenograft studies. Dimeric conjugates using the Thiobridge technology (Abzena) has helped to maintain binding affinities.

## 5. Peptide–Drug Conjugates

Peptides (~1–3 kDa) have even faster penetration properties and a more rapid elimination rate compared to the scaffolds and antibodies described above. Their attractive synthetic manufacturing and ability to incorporate stabilising non-natural residues/chemical moieties at ease leads to many exploitable features as drug-conjugates.

### 5.1. Pentarin–Drug Conjugates

Small peptides are known natural receptor ligands and researchers have gone on to develop synthetic derivatives that have discovery and manufacturing advantages. Peptide display has enabled the rapid isolation of binders through many innovative approaches. The pentarin (pen = penetrate, tar = target) platform consists of small peptides (<2 kDa) that can be made into pentarin-drug conjugates (PDC), developed by Tarveda (formerly Blend) Therapeutics [82]. Their lead compound is PEN-221, a somatostatin receptor-2 (SSTR2) targeting octreotide peptide analogue coupled to DM1 maytansine. SSTR2 is expressed on neuroendocrine tumours, including small cell lung cancer. Other payloads (auristatin E and carbazitaxel) were screened and found to be less effective. DM1 conjugated to the C-terminal side-chain of the disulfide-cyclised Tyr^3^-octreotate was selected as PEN-221, was selective for the SSTR2 receptor with 22 pM affinity, rapidly internalised and a cytotoxic potency of 136 nM on NCI-H524 cells [82]. Interestingly, a more labile disulfide linker as opposed to a hindered disulfide was more efficacious despite being more stable in vivo. Tumour uptake of DM1 was seen to occur rapidly upon dosing, peaking at 2 h. Remarkably, two doses of 2 mg/kg PEN21 were enough to cure H524MD lung cancer tumours (SSTR2-expressing), whereas two doses of 1 mg/kg were needed to cure HCC33 tumours. PEN221 is now in phase 1 clinical trials for SSTR2-expressing tumours [83]. The follow-up compound is PEN866, a PDC carrying the SN38 payload targeting the heat-shock protein chaperone HSP90, up-regulated in many cancers, is currently recruiting for a phase 1/2a clinical trial for solid cancers (colon, small cell lung and sarcoma) sensitive to topoisomerase I inhibitors [84]. 

### 5.2. ‘Bicycle’–Drug Conjugates

Bicyclic peptide (‘bicycles’) can be discovered by phage display techniques using peptide libraries with three fixed cysteines cyclised with a reagent such as TBMB. Less rigid than monocyclic peptides, higher affinities and increased stability can be obtained [85]. Technology discovered and developed by Heinis and Winter [86] is now being commercialised by Bicycle Therapeutics Ltd., including Bicycle-Drug Conjugates (BDCs). A bicyclic peptide recognising the MT1-MMP (human matrix metalloprotease 14) with an affinity of 1.5 nM *K*_D_ was discovered by phage display and chemically synthesised (incorporating some non-natural amino acids to increase stability) to include a DOTA chelating group for gadolinium PET imaging. MT1-MMP is overexpressed in multiple cancers including triple negative breast, non-small cell lung, and soft tissue sarcoma. Rapid tumour accumulation was seen within 1 h, with little uptake elsewhere except for kidney and bladder (renal clearance route) [87]. BT1718 is a BDC based on this bicyclic peptide carrying a DM1 payload via a hindered disulfide linker. A number of different linkers were screened before the final candidate was selected. It has rodent/cynomolgus species cross-reactivity and plasma stability of more than 20 h. Conjugation of the payload results in BT1718 with an affinity of 3 nM. In a variety of high-expressing MT1-MMP NSCLC models, efficacy was seen at 3 and 5 mg/kg BDC given twice-weekly for two-four weeks. Complete cures were seen at 10 mg/kg. Similar results were seen in high-expressing MT1-MMP patient-derived models, all with good tolerability as measured by body weight [88].

### 5.3. RGD Peptide–Drug Conjugates

Other key developments have involved conjugates of cytotoxic agents with the RGD-peptides (Arg–Gly–Asp) targeting integrin receptors (which play a key role in cell adhesion) over-expressed in many solid cancers. The RGD motif was recognised as the minimal recognition sequence that bind to α_v_β_3/5_ receptors, which also retain the ability to be internalised. Similar chemistry concepts have been appliedto the linker-payload and attachment. Many RGD-paclitaxel conjugates have been described with IC_50_ values ranging from 1–100 nM, with tumour cure efficacies at a well-tolerated 36 mg/kg given frequently (twice/week). Similar potencies have been observed for RGD-doxorubicin and RGD-platinum based conjugates and many more have been described, including MMAE-based conjugates [89].

## 6. Small-Molecule–Drug Conjugates (SMDCs)

### 6.1. CA-IX Ligand–Drug Conjugates

The smallest format of drug conjugate would be those containing a small chemical moiety as the targeting ligand. Examples of this include the approved anti-glaucoma drug acetazolamide (AAZ), a ligand for the tumour and hypoxia-associated target carbonic anhydrase IX (CA-IX). Here, SMDCs with a DAR of one using two payloads were evaluated [90]. The targeting ligand itself enabled rapid tumour targeting with significant uptake (13.4% of the injected dose per gram of tissue, higher than some antibodies’ peak uptake) and contrast within 1 h. An AAZ-Duocarmycin conjugate only showed modest tumour growth inhibition, but the AAZ-DM1 was tumouristatic and well tolerated for up to 20 days if administered every day for 7 days (70 nmol of DM1 per dose). The lack of cures was attributed to the moderate affinity (~12 nM) which could be overcome by increasing affinity by dimerization. CA-IX does not significantly internalise, so the linker-payload strategy was a disulfide-based extracellular release mechanism, which also may account for the reduced potency compared to standard internalising ADCs. A higher affinity bivalent AAZ-DM1 drug conjugate, that demonstrated practically no dissociation and better tumour retention was more efficacious in vivo with 33% cures when dosed for 7 sequential days at 35 nmol [91]. Higher doses were not well tolerated.

A high affinity AAZ-based CA-IX targeting SMDC (DAR1) was compared directly to a corresponding ADC (DAR 2) using the vcMMAE payload [92]. An exceptionally high peak uptake (~40% ID/g tumour) and specificity ratio (~100:1) was seen for the SMDC, with superior tumour penetration. Comparing similar payload dosing, the ADC was more efficacious than the SMDC but a significant affect was seen with a non-binding ADC. The non-binding SMDC was ineffective demonstrating clear specificity with the smaller format and a possible slow-release effect of the ADC format. More rapid payload release to match the pharmacokinetics of the SMDC may improve its efficacy.

### 6.2. PSMA Ligand–Drug Conjugates

Endocyte have been innovating and developing SMDCs for a long time. They have published extensively on folate receptor (expressed on ovarian cancers) targeting vinca alkaloids and tubulysin payload conjugates. 

Until recently, their lead product candidate was EC1456, a folate-tubulysin SMDC. However, in June 2017, the clinical development of EC1456 was discontinued due to a lack of efficacy and focus was switched to the development of EC1169, a DUPA conjugate of tubulysin B hydrazide. DUPA is one of the highest affinity small molecular ligands of prostate specific membrane antigen (PSMA) [93], and is highly expressed in taxane-exposed metastatic castrate-resistance prostate cancer patients. As of July 2017, a phase I trial with EC1169 was still recruiting and top-line data was expected at the end of 2017. However, this product candidate does not appear in Endocyte’s pipeline or feature in their most recent investors’ presentation (Jeffries conference, 2017) where CAR-T and targeted radiotherapy has taken prominence. Very little has been published on EC1169, although a recent poster at ESMO in 2017 presented Phase 1b data showing that 6.5 mg/m^2^ was well tolerated [94]. 

An extension of this idea was described by Kumar et al. [95] building a PMSA-targeting SMDC on a PEG scaffold that also incorporated a Gadolinium PET imaging agent; a theranostic small molecule drug conjugate (T-SMDC). Tumour-specific uptake was seen 1 h after administration with high kidney uptake. Cell-killing specificity was also demonstrated in vitro but no absolute potency values were shown for this moderate affinity binding agent (*K*_D_ ~187 nM).

### 6.3. Folate Ligand–Drug Conjugates

Similar examples include vintafolide and etarfolatide, also developed by Endocyte. Both bear a folate analogue for targeting of cells overexpressing the folate receptor. Etarfolatide is then linked to a polydentate ligand, chelating an atom of technetium-99m which can be used as an imaging agent to identify folate receptor expressing tumours. This is used in conjunction with vintafolide, which contains a folate analogue and a vinblastine payload joined by a dithiol cleavable linker. These SMDCs are currently in phase 2b testing against non-small-cell lung carcinoma and phase 3 testing for platinum-resistant ovarian cancer. However, an ovarian cancer study was abandoned in 2014 due to failure to improve survival [96,97]. Glutamic acid urea derivatives, targeting PSMA, mainly used for Tc-99m imaging [98] and via a dendrimer linker to methotrexate (MTX) have also been reported [99]. 

### 6.4. Phosphatidylserine Ligand–Drug Conjugates

Other new tumour-associated ligands that are being developed as SMDCs include phosphatidylserine (PS), which is abundant in the tumour microenvironment. Molecular Targeting Technologies (MTTI) has licensed a technology platform developed at the University of Notre Dame based on zinc(II) dipicolylamine (ZnDPA) coordination complexes that specifically target PS. The lead product is ZnDPA conjugated to the topoisomerase I inhibitor SN-38 through a proprietary enzyme cleavable linker [100]. 

### 6.5. Asialoglycoprotein Receptor Ligand–Drug Conjugates 

Asialoglycoprotein receptor (ASGP-R) belongs to the C-type lectin family and exhibits high affinity for carbohydrates specifically galactose and *N*-acetylgalactosamine (GalNAc). ASGP-R is abundantly expressed in hepatocytes and is an attractive target for receptor mediated drug delivery. Recent published work has described ASGP-R targeted conjugates with both doxorubicin and paclitaxel [101]. The resulting conjugates displayed high affinity to the target but the doxorubicin conjugates were less toxic in vivo than drug alone. 

## 7. Discussion and Conclusions

As illustrated by the many and varied array of small formats (Figure 1; Table 1), there is a lot of activity and innovation in this space. As a relatively new area, much of it does not get covered in mainstream ADC reviews. It is clear that small-format targeting vehicles are re-emerging commercially after the promising review by Hollinger et al. some 20 years [102]. A recent pipeline analysis in Nature Biotechnology supports this [103]. Included in this is the rise and commercial validation of alternative binding scaffolds which address the manufacturing and stability issues often encountered by antibodies and derived fragments, so the attachment of effector functions such as cytotoxic payloads is a natural choice. Other scaffolds that did not really find a place in the body of this review include one approach by CytRx who are developing a prodrug payload that attaches to human serum albumin (HSA), to form a drug-conjugate in vivo. Aldoxorubicin (INNO-206), reacts with Cys-34 of HSA. This albumin–drug conjugate allows the passive uptake of doxorubicin and tumour-selective release due to the acid-labile linker. This reduces cardiac toxicity side effects and is in Phase 3 clinical trials for soft tissue sarcoma [104]. Another example would include receptor-specific ligands such as Fibroblast growth factor (FGF). An FGF-vcMMAE conjugate (~15 kDa) for breast, lung and gastric cancer demonstrated nanomolar potency dependent on receptor expression [105]. There could be many more examples of these to come but the proprietary position would be unclear.

**Figure 2:**
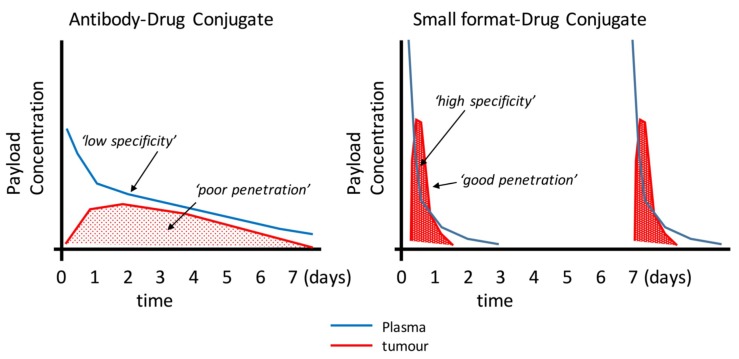
Schematic illustration of the potential mechanistic and pharmacokinetic differences between an ADC (e.g., from [110] and small format-drug conjugate. Many reports have demonstrated the uptake and clearance properties of large and small targeting agents. This figure proposes how smaller formats my bring certain advantages such as faster plasma clearance and improved penetration. Smaller formats may require more frequent doses as shown here.

Small-format drug conjugates at first look seem an ideal solution for solid tumours—better penetration, access to hidden epitopes otherwise inaccessible to larger antibodies, and lower non-target tissue exposure (especially Fc-receptor binding that leads to thrombocytopenia and neutropenia in ADCs) [106] are over-whelming benefits that deserved to be exploited, but these improvements come at the expense of lower absolute uptake, a seemingly conflicting property. Small molecule ligands (e.g., AAZ or peptides) overcome this somewhat due to their high peak uptake afforded by their rapid extravasation properties. Therefore, to translate the promising features of small formats and antibody fragments into patient benefits will require a lot of research and development. It’s clear that this area is emerging with very little in vivo data available, especially for the alternative scaffolds. Where antibody fragment examples exist, small-format drug conjugates are generally inferior to ADCs due to lower overall payload delivery (e.g., Fabs and some scFv approaches). Notable examples are the diabody–drug conjugates described by Seattle Genetics [47] or highly-loaded scFv–drug conjugates described by Antikor Biopharma [62,63] where the efficacy is starting to be comparable to ADCs. Could it be that the improved penetration is having an effect despite the more rapid elimination (Figure 2)? However, the patient value in terms of improved clinical tolerability and therapeutic index has yet to be shown. The topic of payload delivery format is indeed contentious. One key paper by Hinrichs et al. [107] supports the idea that the overall tumour exposure of the payload drives the efficacy as giving the ADC as an equivalent fractionated dose is as effective as fewer large doses, with better tolerability. This idea does not quite align with the concept of high peak dosing being favourable to drive tumour penetration, a key differentiating benefit of smaller format ADCs. However, it was clearly shown that in order to achieve improved penetration of trastuzumab-emtansine, effectively raising the peak concentration using free trastuzumab did improve tumour penetration for many of the arguments described above [37]. To further complicate our understanding of the whole concept of ‘targeting’, Neri’s comparison of the two extremes (small and large) of drug conjugates [92] indicated that a significant part of the ADC property was the slow-release of the payload. Although surprising, if one can administer more payload systemically to drive the improved penetration and efficacy without eliciting adverse effects, then perhaps we should not be comparing conjugates with the same quantity of payload. Perhaps it’s more informative to compare efficacy at the maximum tolerated dose. This would be more clinically-relevant. 

The importance of the payload cannot be under-estimated. There is a trend moving away from microtubule-inhibiting (MTI)-based payloads towards the more potent DNA-damaging agents that also act on non-proliferating cells. Around 40% of MTI ADCs have been discontinued due to lack of efficacy [3]. But, increasing the potency of the payload may not be the solution because DNA-damaging agents may have cumulative toxicity (unlike MTIs). Consequently, companies like Immunogen are ‘tuning down’ their payloads in order to find a ‘sweet spot’ leading to a larger therapeutic window [5]. Without more pre-clinical data in animal models and clinical efficacy/tolerability data, it is too early to say which will be the best small format for payload delivery. This may vary with indication, with tumours that have particular penetration challenges (e.g., pancreatic) benefitting more from such an approach, so strategic development would have to include data where formats were compared in large volume or stromal-dense tumour models in order to select the most promising candidate. Efficacy is only half of the equation: better pre-clinical indicators of toxicity will be needed in order to describe a true therapeutic index that gives clinicians hope that enough of the therapeutic can be administered to humans to reach all parts of the tumour. This confirms the general consensus in oncology drug development, the eventual therapeutic window will need to be demonstrated in man and this will have required a careful choice of indication, target, and payload.

## Figures and Tables

**Table 1:** Summary of the various small-format drug conjugates in clinical and pre-clinical development.

Small Format Carrier Size (kDa)	Small Format Carrier	Target	Payload	Status [Reference]	Commercial Organisation (If Any)
~0.2	*N*-Acetyl-galactosamine	Asialo-glycoprotein receptor	doxorubicin, paclitaxel	Pre-clinical R&D [101]	
~0.2	Acetazolamide	Carbonic anhydrase IX	DM1, duocarmycin	Pre-clinical R&D [90,91,92]	Philogen SA
~0.3	DUPA	PSMA	Tubulysin	Pre-clinical R&D [94]	Endocyte Inc.
~0.4	Folate	Folate receptor	Vinca alkaloid, Tubulysin	Pre-clinical R&D [96,97]	Endocyte Inc.
~0.7	Zinc dipicolylamine	Phosphatidyl-serine	SN38	Pre-clinical R&D [100]	
~1	RGD-based peptides	Intergrins	Various: doxorubicin, vcMMAE, paclitaxel	Pre-clinical R&D [89]	
1.5–2	Bicycle (bicyclic peptides)	Matrix metallo-protease 14	DM1	BT1718 is in Phase 1/2a clinical trials [86,87,88]	Bicycle Therapeutics Ltd.
~3–5	Pentarin	Somatostatin receptor	DM1	PEN-221 is in Phase 1/2a clinical trials [82,83,84]	Tarveda Inc.
~3.5–5	Cystine knots	Integrin, Matrix metallo-protease 2	Gemcitabine, MMAF, Cis-platin		
5–6.5	Affibody	HER2	Idarubicin, vcMMAE	Pre-clinical R&D [67,68,69]	Affibody
7	Nanofitin (sac 7d)	ND *	ND *	Pre-clinical R&D [108]	Affilogic
~10–11	Centyrin	EGFR	vcMMAF	Pre-clinical R&D [72]	Janssen R&D LLP
~12	VH (like) domains	ND *	ND *	Pre-clinical R&D	Crescendo Biologics Ltd.
12–14	Affimer	ND *	ND *	Pre-clinical R&D [109]	Avacta PLC
~15–18	DARPIn	EpCAM	MMAF	Pre-clinical R&D [78,79]	Molecular Partners AG
~15	Abdurin	ephA2	vcMMAE	Pre-clinical R&D [80]	IRBM
~25–27	Single-chain Fv	Various: HER2, CEA, PLAP, Fibronectin, Tenascin-C	Adriamycin, photosensitisers, Infra-red dyes, MMAF, vcMMAE, doxorubicin, cemadotin, dolostatin-10	Pre-clinical R&D [56,57,58,59,60,61,62,63]	Antikor Biopharma Ltd.; Philogen SA
~55–60	Diabody	CD30	MMAF	Pre-clinical R&D [47,48]	Seattle Genetics Inc. Avipep
~50	Fab	Various: TROP2, CD20, HER2	Doxorubicin, vcMMAE, Auristatin	Pre-clinical R&D [40,41,42,43,44,45]	Abzena
~80	SIP-Small immunoprotein	Fibronectin, Tenascin-C	Cemadotin, DM1	Pre-clinical R&D [49,50,51,52,53,54]	Philogen SA

* ND—Not disclosed.
